# Case report: Recurrent catatonia in a patient with 17p13.3 microduplication syndrome

**DOI:** 10.3389/fpsyt.2025.1607003

**Published:** 2025-06-26

**Authors:** Ilya Querter, Nika Schuermans, Nele Van de Velde, Cisse Geleyn, Annelies Dheedene, Kurt Audenaert, Chris Baeken, Bert Callewaert, Gilbert Lemmens

**Affiliations:** ^1^ Department of Head and Skin - Psychiatry, Ghent University, Ghent, Belgium; ^2^ Department of Psychiatry, Ghent University Hospital, Ghent, Belgium; ^3^ Center for Medical Genetics, Ghent University Hospital, Ghent, Belgium

**Keywords:** 17p13.3 microduplication syndrome, catatonia, depression, case report, genetic assessment

## Abstract

Catatonia is a clinically significant syndrome with various etiologies, including genetic factors, that are increasingly recognized. We present a case of recurrent catatonia associated with 17p13.3 microduplication syndrome in a 47-year-old woman with a long-standing history of recurrent depressive episodes. At age 44, she experienced her first episode with psychotic and catatonic features, which required hospitalization. Over the next three years, she had four additional catatonic episodes. Four years after her initial presentation, she was diagnosed with 17p13.3 microduplication syndrome. This case emphasizes the importance of considering genetic testing for patients with recurrent catatonia, particularly those with a comorbid developmental disorder. Given the limited number of cases of 17p13.3 microduplication syndrome reported in the literature, we share these findings to encourage prompt genetic assessment in similar presentations. Clinicians treating patients with catatonia should recognize the prevalence of medical, and particularly genetic, disorders that increase susceptibility to catatonia. Conversely, clinicians working with patients who have genetically based neurodevelopmental syndromes should be aware of the challenges in diagnosing and treating catatonia. By identifying catatonia in this patient population, prompt and targeted interventions that may significantly reduce the disabling effects of catatonia can be initiated. This case also expands the known phenotypic spectrum of 17p13.3 microduplication syndrome and contributes to understanding the genetic factors involved in catatonia, though further research is needed to clarify this association.

## 1 Introduction

Catatonia is a neuropsychiatric syndrome characterized by a distinct set of psychomotor disturbances (1, 2). Historically regarded as a subtype of schizophrenia, catatonia is now understood to manifest across a broad range of both psychiatric and medical conditions (2). Reports indicate that catatonia is present in over 10% of patients with acute psychiatric disorders (2), with its prevalence varying among different psychiatric conditions, particularly showing higher rates in affective disorders (3). This condition can lead to numerous medical complications (such as malnutrition, ulcers, contractures, rhabdomyolysis, and thrombosis) and may progress to malignant catatonia—a severe form with an estimated mortality rate of approximately 20% (4). Therefore, it is important to consider underlying medical conditions that may present with catatonic features (e.g., encephalomyelitis or diabetic ketoacidosis), as these require additional treatment beyond the initial management of catatonia (1). Catatonia is typically treated with benzodiazepines, ECT, NMDA receptor antagonists, or a combination of these therapies (4).

A wide range of psychiatric and medical conditions, such as infectious, autoimmune, metabolic, and genetic disorders, are known to be associated with catatonia (4). When catatonia is secondary to an underlying medical condition, it tends to be less responsive to conventional treatments specific to catatonia (e.g benzodiazepines), pointing to the necessity to thoroughly investigate potential etiological factors, particularly when there is a diminished response to standard therapeutic interventions (4). This case report details recurrent catatonia in a patient with a 17p13.3 microduplication syndrome. This syndrome is exceedingly rare, with only about 40 cases documented globally (5). Duplications on the 17p13.3 chromosomal region, impacting genes such as *YWHAE*, *CRK*, and *PAFAH1B1*, are established risk factors for various neuropsychiatric conditions, including intellectual disability, autism spectrum disorder, and behavioral problems (6, 7). This report expands the clinical presentation of 17p13.3 microduplication syndrome and highlights the importance of genetic testing in patients presenting with recurrent catatonia.

## 2 Case description

### 2.1 Initial presentation

A 47-year-old woman was admitted to the psychiatric ward of a general hospital due to a catatonic state. She had been referred by her outpatient psychiatrist, whom she had seen once approximately one month prior to admission. During that initial consultation, the patient presented with prominent anxiety and disorganized speech, as she struggled to articulate her distress and conveyed her experiences in a fragmented and chaotic manner.

Over the following weeks, her condition deteriorated markedly. In the days leading up to admission, she refused both her prescribed medication and food, and became increasingly mute. Due to escalating concerns—including suicidal ideation and a severe decline in daily functioning—she moved in with her sister. When this arrangement became unsustainable for the family, she was referred to the emergency department.

Upon arrival, the patient presented as withdrawn, avoidant of eye contact, and largely non-responsive. According to her sister, she frequently expressed remorse for unspecified “irreversible” actions and made suicidal statements, such as “I might as well end it.” During her hospitalization, she remained socially withdrawn, interacted minimally with other patients, and participated in ward activities only when prompted by staff.

Her psychomotor behavior was striking. She often remained immobile in fixed standing or sitting positions for prolonged periods (catalepsy). Attempts to initiate movement—for instance, asking her to sit down when standing—were met with significant resistance, suggestive of negativism and rigidity. This motor inhibition was accompanied by pronounced ambitendency, manifested in hesitant and indecisive gestures.

At the peak of the catatonic episode, the patient was mute or responded only with faint whispers. When verbal communication occurred, it was characterized by verbigeration (repetition of fragmented or stereotyped phrases), and perseveration, with a persistent return to themes of guilt and social persecution. Furthermore, a psychotic delusion of guilt that she had committed a murder became increasingly pronounced. No manic symptoms were observed.

According to the patient and her family members, there were no apparent stressors or significant changes preceding the onset of her symptoms. The patient had however a history of several depressive episodes throughout her adult life. At the age of 44, the patient experienced a first major depressive episode with psychotic and catatonic features (retardive type (2)), requiring a psychiatric admission. Treatment with lorazepam (4x1mg daily) resulted in a gradual improvement of her symptoms. After approximately three days, the patient began to communicate verbally again, and her motor behavior became more fluid and responsive.

As maintenance therapy since her first catatonic episode, she was prescribed a combination of Nortriptyline (100 mg daily) and Lorazepam (4x1 mg daily). Despite this daily treatment, she experienced four additional catatonic episodes over the next four years, all requiring hospitalization.

After the first episode, a gradual tapering of lorazepam was initiated; however, reductions below 4 mg/day repeatedly triggered a re-emergence of catatonic symptoms, which could be attributed either to a rebound effect or to a more chronic course of the illness—this distinction remains unclear. Following the second episode, a deliberate decision was made to continue lorazepam as maintenance therapy, both due to concerns about relapse and the previous difficulties encountered during dose reduction.

Catatonic symptoms were consistently prominent, accompanied by mild psychotic features, while the affective component became less pronounced during subsequent episodes. Each episode responded well to an increased dosage of Lorazepam (up to 16mg daily), leading to rapid remission of symptoms. Tapering was initiated cautiously at 1 mg every 48 hours from the point of remission. If catatonic symptoms re-emerged, the process was delayed, returning to the lowest effective dose and a slower tapering schedule. The treatment was well tolerated. Notably, electroconvulsive therapy (ECT) was not required at any point during this period.

### 2.2 Presentation during inter-episode periods

A similar presentation across the different episodes was consistently observed, with each recurrence characterized by a nearly identical pattern of catatonic and psychotic symptoms. Between episodes, the patient exhibited no more than mild depressive symptoms, and occasional paranoid ideation was reported—for example, expressing the belief that “the neighbors are gossiping about me”. Psychomotor symptoms were not observed during the inter-episode periods. Importantly, there were no clinical indications of (hypo)manic episodes throughout the course of her illness.

### 2.3 Medical history

The patient’s prior diagnostic history includes a neuropsychological evaluation conducted at age 34, which revealed a fluid IQ of 88 and a crystallized IQ of 103, culminating in a total IQ of 95 with a notably uneven cognitive profile. The Kaufman Adolescent and Adult Intelligence Test (KAIT) was used for this assessment, and the patient demonstrated difficulties with verbal expression (the verbal expression was characterized by disorganized and unfocused speech and the patient often responded impulsively and prematurely, indicating challenges with self-inhibition and goal-directed verbal output). In the preceding year, a DSM-5 diagnostic interview was performed to evaluate for autism spectrum disorder, prompted by longstanding difficulties in social interaction, reduced nonverbal communication (e.g., poor eye contact and limited facial expressiveness), and a restricted social network. However, the assessment did not reveal clinically significant findings consistent with an autism spectrum diagnosis.

There was no significant family history of psychiatric or medical disorders, and no consanguinity was reported. The patient was born 3 weeks prematurely, but her perinatal and neonatal courses were uneventful. Her speech and language development were normal for her age group. Motor development was slightly delayed, with walking not achieved until approximately 18 months of age. She underwent motor skills rehabilitation during the 2nd and 3rd grades. She was diagnosed with amblyopia during childhood. She recalls being taller than her peers in childhood and exhibited motor clumsiness. Although she had difficulties with academic performance, she ultimately completed secondary school and worked as a caregiver in a nursing home until the onset of her catatonic episodes at age 44. Given her increased vulnerability to stress and recurring catatonic episodes, a return to professional work has not been possible. Instead, meaningful structure is provided through volunteer activities and a closely monitored support network. The patient lives alone and manages basic daily tasks such as cooking and administrative paperwork, she relies on regular support from family, household assistance, and public transport due to benzodiazepine-related driving restrictions.

### 2.4 Clinical examination

On physical examination at age 47, the patient’s height was 169 cm, and she exhibited macrocephaly (head circumference of 59 cm, >P99), as well as multiple skeletal abnormalities, including thoracic kyphoscoliosis, pes cavus, hallux valgus, splayed feet, bilateral elbow extension deficits, and hypermobility of the proximal interphalangeal joints. Additionally, scattered atypical naevi were observed on her trunk. Facial features included downslanted palpebral fissures, a broad and high nasal bridge, anteverted nares, a long philtrum, thin lips, downturned corners of the mouth, micrognathia, a pronounced highly arched palate, and poor dental quality.

## 3 Timeline

**Figure 1:**
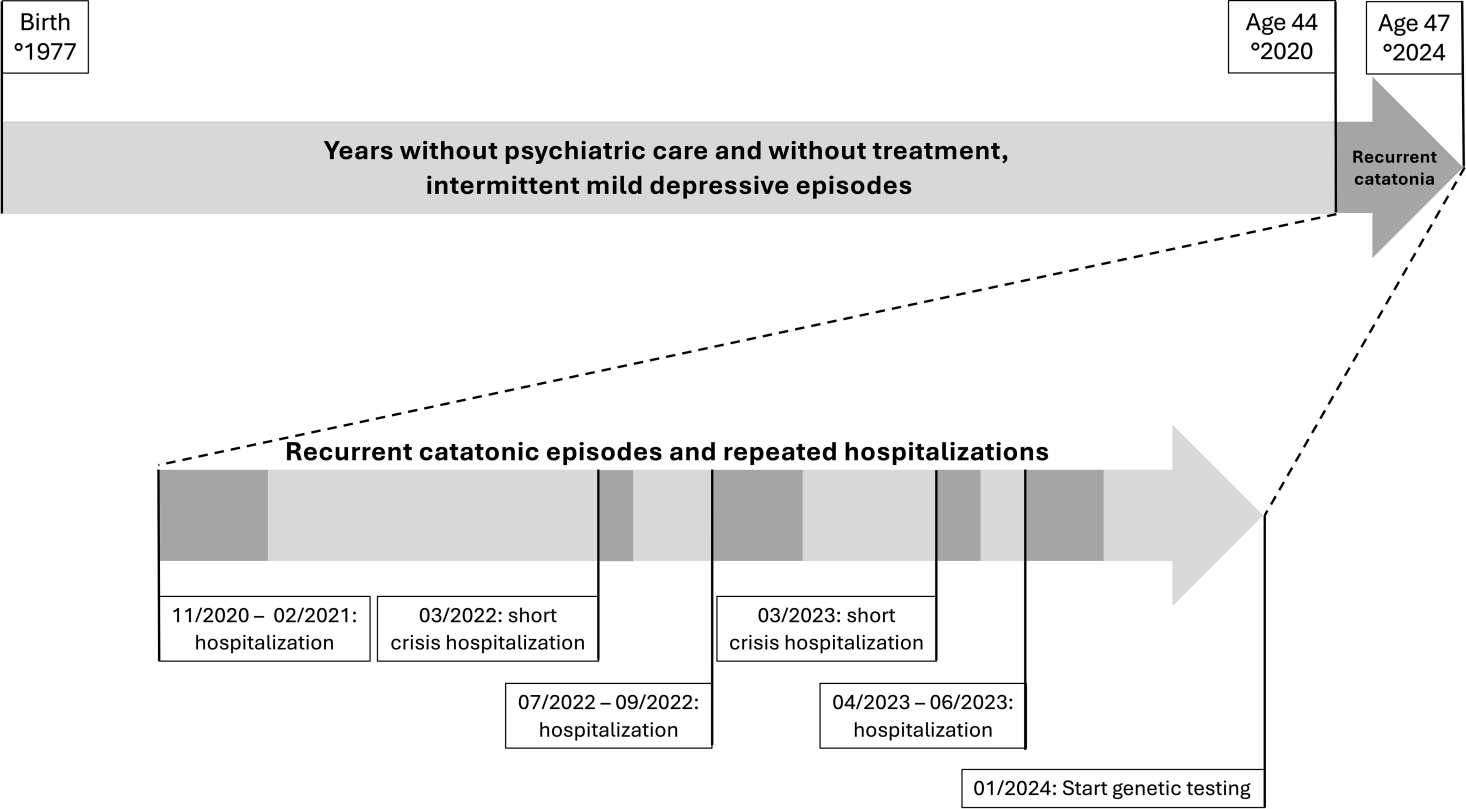
Timeline of the clinical course in a patient with 17p13.3 microduplication syndrome and recurrent catatonia.

An overview of the clinical course, including episodes, is presented in **Figure 1**.

## 4 Diagnostic assessment

Based on the presenting clinical symptoms, the patient met the DSM-5 classification criteria for catatonia, and symptom severity was quantified using the Bush-Francis Catatonia Rating Scale (most recent episode rating: score 20). Symptomatic improvement following a Lorazepam challenge test provided further confirmation of the catatonia diagnosis.

The recurrent pattern of catatonia observed in this case is noteworthy. In the literature, the term periodic catatonia (PC), though not officially recognized as a distinct subtype, has been used to describe rare presentations of recurrent catatonic episodes with remissions in between (8). In our patient, psychomotor remission was observed between episodes; however, she remained on maintenance treatment. Previous attempts at tapering resulted in a re-emergence of catatonic symptoms, raising the question of whether this reflects a rebound phenomenon related to dose reduction, or instead indicates an underlying chronic form of catatonia with a remitting course.

Subsequently to the catatonia diagnosis, an extensive diagnostic work-up was conducted to explore underlying medical conditions for the catatonic episodes. There were no clinical or biochemical indications of infectious, autoimmune, or metabolic causes. Additionally, brain MRI and EEG examinations yielded no abnormal findings. No relevant medication or substance use was identified. Consequently, the patient was diagnosed with major depressive disorder with psychotic and catatonic features based on the clinical presentation and medical history.

Given the recurrent and severe nature of the catatonia, as well as the presence of isolated mild gross motor delay and atypical clinical findings with facial and skeletal dysmorphisms, the patient was referred to a genetics center for further evaluation. Molecular karyotyping was performed by shallow whole genome sequencing at an average 1x genome coverage on an NovaSeq6000 sequencing instrument as described earlier (9). This analysis identified a ~1 Mb duplication on chromosome 17, compatible with 17p13.3 microduplication syndrome (sseq[GRCh38] 17p13.3(1065001_2130000)x3). The duplication was absent in both her parents. The duplication encompasses 23 protein coding genes including the *YWHAE* gene.

Due to the presence of a neurodevelopmental phenotype combined with dysmorphic facial features and skeletal anomalies, concomitant exome sequencing was performed followed by trio analysis of a virtual gene panel including genes associated with developmental delay and epilepsy. This analysis did not identify other (likely) pathogenic variants.

## 5 Discussion

### 5.1 Etiopathogenesis of catatonia

Catatonia symptoms show substantial heritability, with psychomotor retardation and excitement exhibiting moderate familial patterns, while mutism and rigidity demonstrate a high degree of familial heritability (10, 11). Leonhard’s findings indicated that periodic catatonia exhibits familial clustering, contrasting with stable (systematic) catatonia, which shows minimal familial aggregation (11, 12). The lifetime morbidity risk for catatonia among first-degree relatives of individuals with periodic catatonia is approximately 27% (3, 11).

Rare copy number variants (CNVs) are enriched in patients with intellectual disabilities who develop catatonia (11). Notably, many of these CNVs are located in genes associated with gamma-aminobutyric acid (GABA) and glutamatergic signaling pathways, including *SHANK3*, *NRXN1*, *NLGN2*, *KIDINS220*, and *SORCS1*. This is in accordance with the prevailing hypothesis for catatonia’s pathophysiology, showing a dysregulated GABA and glutamate signaling with potentially involving GABA hypoactivity and/or glutamate hyperactivity (13).

Breckpot el al analyzed 15 patients with neurodevelopmental delays and catatonia. CNVs were classified as causal when they either completely overlapped with chromosomal syndromes associated with catatonia or contained catatonia-related genes identified as dosage-sensitive (14). CNVs were deemed likely causal if they affected genes or critical regions linked to chromosomal syndromes known to play a role in intellectual disability or psychiatric disorders, although not directly associated with catatonia (14).

Clinicians treating patients with catatonia should recognize the prevalence of medical, and particularly genetic, disorders that increase susceptibility to catatonia. Some of these underlying disorders, such as creatine deficiency, are treatable and can result in improved outcomes compared to psychiatric treatment alone. Conversely, clinicians working with patients who have genetically based neurodevelopmental syndromes should be aware of the challenges in diagnosing catatonia due to symptom overlap (e.g. mutism and stereotypies) (15). Clinicians should have a high index of suspicion for catatonia when there is a sudden decline in motor activity, speech, or behavior, even if the patient already has limited expressive abilities (16).

### 5.2 17p13.3 microduplication syndrome

The 17p13 chromosomal region is known for its genomic instability with a susceptibility to submicroscopic rearrangements, including deletion and duplication events. The 17p13.3 microduplication syndrome, a rare genetic condition with only about 40 reported cases globally, is located within the region where deletions cause Miller-Dieker syndrome, and presents a broad spectrum of phenotypic features. Critical genes involved in the Miller-Dieker phenotype include *YWHAE* and *PAFAH1B1* (5).

Two classification systems are commonly used in the literature to categorize 17p13.3 microduplication syndrome. Bruno et al. proposed to divide the duplication into Class I and Class II based on the involvement of key genes. Class I duplications include *YWHAE* (encoding 14-3-3ϵ) but not *PAFAH1B1*. Class II duplications always involve *PAFAH1B1*, sometimes alongside *YWHAE* (5, 6). In contrast, Curry et al. classified the duplications into three categories: Group 1 (small telomeric duplications involving *YWHAE*), Group 2 (larger duplications spanning most of 17p13.3), and Group 3 (small centromeric duplications including *PAFAH1B1*) (7).

A distinct phenotype is most commonly observed in individuals with larger duplications that encompass both the *YWHAE* and *PAFAH1B1* genes. These individuals often display a characteristic facial profile and structural brain anomalies. Autism spectrum disorders are reported in approximately one-third of affected individuals, particularly among those with duplications of *YWHAE* and adjacent genes such as *CRK*. The typical neurobehavioral phenotype is generally seen in cases with larger duplications (7).

### 5.3 Remarks and conclusions

The presented patient’s exact CNV is designated as sseq[GRCh38] 17p13.3(1065001_2130000)x3 dn, placing her in Class I as defined by Bruno et al. or in Group 2 as defined by Curry et al. This duplication spans several genes, including *ABR*, *BHLHA9*, *YWHAE*, *CRK*, *PITPNA* and *HIC1* that are known to be dosage sensitive (pTriplo score>0.6) (17).

Duplications on 17p13.3 affecting *YWHAE*, *CRK*, and/or *PAFAH1B1* have been identified as susceptibility factors for intellectual disability, hypotonia, autism spectrum disorders, behavioral disturbances, brain malformations, dysmorphic facial features, and limb anomalies (6, 7). SNPs in *YWHAE*, encoding 14-3-3ϵ, have been associated with increased susceptibility for major depressive disorder, bipolar disorder and schizophrenia (18–22). 14-3–3 proteins are highly expressed in the brain and contribute to the modulation of synaptic plasticity and neuronal development (23). An increased expression of YWHA genes was observed during conversion to psychosis in a cohort of patients at ultra-high risk for psychosis. This upregulation suggests a potential role for YWHA in the pathophysiology of early psychosis (23).

Our patient presented with mild gross motor delay, facial dysmorphism and skeletal features, though IQ was within the normal range and no brain malformations or limb anomalies were observed. She did not meet the criteria for a diagnosis of autism spectrum disorder. The primary and most debilitating symptom for our patient was recurrent catatonia, which significantly impaired her daily functioning. Despite having been able to participate effectively in society throughout her life, the onset of catatonic episodes brought substantial disability.

It remains essential to critically assess the etiological relevance of each copy number variant (CNV) within a patient’s clinical presentation, as CNVs contribute significantly to human genetic diversity. While CNVs can underlie Mendelian or sporadic traits and are associated with complex diseases, they may also exist as benign polymorphic variants without clinical impact (24).

Whether the patient’s catatonic symptoms can be completely attributed to the 17p13.3 microduplication syndrome remains therefore elusive. Since the 17p13.3 microduplication has been reported to be inherited of asymptomatic or mildly affected parents in the literature (7), However, it is important to note that the cases described in the literature involve individuals under the age of 45 (6, 7, 25), making the clinical manifestations in later adulthood unclear—our patient is 47 years old. Furthermore, catatonia can be challenging to diagnose unless specifically and systematically assessed (26).

To determine whether recurrent catatonia could be a clinical manifestation of 17p13.3 microduplication syndrome, we applied the classification algorithm proposed by Breckpot. According to Breckpot’s classification algorithm, this CNV is considered “likely pathogenic” due to the involvement of genes implicated in intellectual disability and psychiatric conditions, though not specifically associated with catatonia (14).

It is noteworthy that, to date, only one other patient with 17p13.3 microduplication syndrome has been reported with catatonic features (14). In this patient, the duplication included the genes *SLC43A2*, *SCARF1*, *RILP*, and *PRPF8*. These are non-critical genes linked to catatonia, intellectual disability or psychiatric conditions. Therefore, this CNV was classified as a variant of unknown significance (14). In contrast, the genetic duplication identified in the present case is considered likely pathogenic, due to the involvement of critical genes associated with neuropsychiatric vulnerability.

For 17p13.3 microduplication syndrome, additional case reports will be essential in determining whether catatonia constitutes a significant clinical feature of this condition. Such reports may help pinpoint which genes within the 17p13.3 region contribute most critically to catatonia susceptibility. The limited literature on this association could be due to the underdiagnosis of catatonia itself (26) or to the possibility that catatonia may predominantly manifest later in life, as noted in previous studies, and thus may not be observed in younger patients (3, 11). Future efforts should focus on genetic testing in patients with recurrent catatonic episodes and on longitudinal clinical follow-up of individuals carrying this microduplication into later adulthood (beyond 45 years), in order to document the potential emergence of catatonic symptoms.

## 6 Patient perspective

Throughout the various catatonic episodes, the patient was informed about catatonia, including an explanation of its neurobiological underpinnings. Despite this, the patient frequently experienced significant embarrassment as the episodes became more frequent. She consistently consented to a series of diagnostic investigations aimed at identifying potential underlying causes.

The diagnosis of the 17p13.3 microduplication syndrome provided substantial clarification for the patient, although the unchangeable nature of the condition was challenging for her to process. Supported by her loved ones, she engaged in an acceptance process and, over time, expressed gratitude for the diagnostic clarity. She was particularly relieved to learn that her condition was due to a *de novo* event, meaning no family members were affected.

## 7 Conclusion

This report presents a new case of 17p13.3 microduplication syndrome with recurrent catatonia, potentially expanding the known phenotypic spectrum of this rare disorder. This case underscores the importance of further exploring the genetic underpinnings of catatonia, and illustrates how rare genetic variants might contribute to its clinical expression. Additional case reports describing unique associations between genetic anomalies and catatonic features will be essential to better understand this relationship.

## Data Availability

The original contributions presented in the study are included in the article. Further inquiries can be directed to the corresponding author.
